# No Correlation Between Childhood Maltreatment and Telomere Length

**DOI:** 10.1016/j.biopsych.2010.02.026

**Published:** 2010-09-15

**Authors:** Daniel Glass, Leopold Parts, David Knowles, Abraham Aviv, Tim D. Spector

**Affiliations:** a Kings' College London, Department of Twin Research and Genetic Epidemiology, St. Thomas' Hospital Campus, Westminster Bridge Road, London SE1 7EH, United Kingdom; b Wellcome Trust Sanger Institute, Hinxton, United Kingdom; c University of Cambridge, Cambridge, United Kingdom; d University of Medicine and Dentistry New Jersey, Newark, New Jersey; e Kings' College London, Department of Twin Research and Genetic Epidemiology, London, United Kingdom

To the Editor:

Telomeres are lengths of repetitive DNA that cap the ends of chromosomes. They protect the ends of the chromosome and shorten with each cell division. Short leukocyte telomere length has been related to a number of age-related diseases (1). In addition, shorter telomere length has been associated with environmental factors such as smoking and lack of exercise (2,3). In a recent issue of *Biological Psychiatry*, Tyrka *et al.* (4) published a report suggesting a link between maltreatment in childhood and telomere shortening in 31 subjects. Individuals who had suffered maltreatment had telomere length .70 ± .24 compared with 1.02 ± .52 in individuals who had not been abused.

We were interested in this plausible theory and decided to investigate these findings in the larger Twins UK cohort. Twins UK is a registry of ∼11,000 British twins that has been shown to be representative of the general population. Twins have answered questionnaires on two occasions relating to maltreatment, be it sexual, physical, or emotional abuse. One questionnaire asked specifically about maltreatment in childhood (2005) and one referred to maltreatment at any time (2008). The specific questions asked were the following: “As a child, were you ever a victim of physical abuse?” “As a child, were you ever a victim of sexual abuse?” “Have you ever been physically abused?” “Have you ever been emotionally abused?” “Have you ever been sexually abused?”

The possible answers were Yes, No, or no answer. We only considered subjects that gave a consistent answer to the physical abuse or the sexual abuse questions in both questionnaires. We felt it further strengthened our phenotype to have corroborative data at two time points.

Mean leukocyte telomere length was derived from the mean of the terminal restriction fragment length by using the Southern blot method on DNA extracted from peripheral leukocytes, as described elsewhere (5). Each DNA sample was analyzed twice. If the difference between the duplicates was >5%, a third measurement was performed, and the mean of the two results <5% apart was taken. The coefficient of variation of the terminal restriction fragment length assay in the study was 1.5%. Telomere length was essentially normally distributed.

There is no significant difference in telomere length between people who have been abused and those who have not been abused (Figure 1, Table 1). The result holds for parametric and nonparametric tests and including or excluding possible confounding factors (age, gender, smoking, and body mass index).

When we looked at individuals who had answered the maltreatment in childhood questionnaire alone, we had a much larger sample size. With 123 cases and 1751 control subjects, there remains no significant difference in the mean telomere length between those subjects who had suffered sexual abuse and those who had not. The mean telomere length in this group is identical in cases and control subjects. This result was unchanged when possible confounders (age, body mass index, smoking status, and gender) were included in the linear model.

We have failed to replicate the findings of Tyrka *et al.* (4), despite a sample size several-fold larger. There are certain methodological differences that may account for this lack of replication. Our telomere measurements were performed with Southern blot as opposed to quantitative polymerase chain reaction used in the previous article. We have, however, previously demonstrated significant changes using these assays with other phenotypes, such as lack of exercise, obesity, smoking, social class, and number of nevi. The assays are significantly correlated and the Southern blot method, although labor intensive, is thought to be the gold standard (6).

The questionnaires to assess abuse used by Tyrka *et al.* (4) and Twins UK are different. We have confidence in our phenotype; by using twins who have reported maltreatment in both questionnaires, we have isolated a robust group of cases for analysis.

Many different environmental factors are known to reduce telomeres, such as cigarette smoking, obesity, lack of exercise, and social class, as well as genes (2,3,7). With a small selected sample such as that used by Tyrka *et al.* (4), rather than the abuse, any of these confounding external factors could actually be responsible for the difference in telomere length they observed or it could have been due to chance.

## Figures and Tables

**Figure 1:**
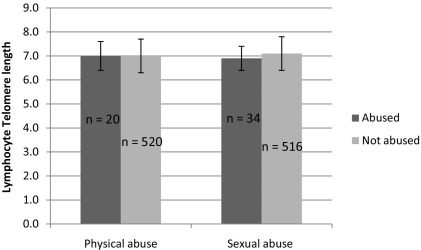
A graph showing no significant difference in mean telomere length in those who had suffered physical or sexual abuse compared with those who had not suffered abuse (*t* test *p* value .61 and .10, respectively). LCL, .

**Table 1:** Telomere Length in Individuals with a History of Abuse Compared with Those with No History of Abuse

	Mean and Standard Deviation of Telomere Length in Subjects with a History of Abuse	Mean and Standard Deviation of Telomere Length in Subjects with no History of Abuse	Number of Subjects (Abused)	Number of Subjects (Not Abused)	Two-Sample *t* Test *p* Value	Mann–Whitney Test *p* Value
Physical Abuse	7.04 (.58)	6.97 (.67)	20	520	.61	.71
Sexual Abuse	6.86 (.53)	7.05 (.66)	34	516	.10	.13
